# Phospho-Proteomics Analysis of Early Response to X-Ray Irradiation Reveals Molecular Mechanism Potentially Related to U251 Cell Radioresistance

**DOI:** 10.3390/proteomes13010001

**Published:** 2024-12-25

**Authors:** Ousseynou Ben Diouf, Antoine Gilbert, Benoit Bernay, Randi G. Syljuåsen, Mihaela Tudor, Mihaela Temelie, Diana I. Savu, Mamadou Soumboundou, Cheikh Sall, François Chevalier

**Affiliations:** 1 Mixed Research Exploration and Diagnosis (UMRED), UFR-Healthy, Iba Der THIAM University of Thies, Thies BP A967, Senegal; oben.diouf@univ-thies.sn (O.B.D.);; 2 UMR6252 CIMAP, Team Applications in Radiobiology with Accelerated Ions, CEA-CNRS-ENSICAEN, Université de Caen Normandie, 14000 Caen, France; 3 Proteogen Platform, US EMerode, CAEN Normandie University, 14032 Caen, France; 4 Department of Radiation Biology, Institute for Cancer Research, Norwegian Radium Hospital, Oslo University Hospital, 0379 Oslo, Norway; 5 Department of Life and Environmental Physics, Horia Hulubei National Institute of Physics and Nuclear Engineering, 077125 Magurele, Romaniadsavu@nipne.ro (D.I.S.)

**Keywords:** bioinformatics, glioblastoma, phosphoproteomics, mass spectrometry, radioresistance, X-rays

## Abstract

Glioblastoma (GBM) is a devastating malignant brain tumor with a poor prognosis. GBM is associated with radioresistance. Post-translational modifications (PTMs) such as protein phosphorylation can play an important role in the cellular response to radiation. To better understand the early cellular activities after radiation in GBM, we carried out a phospho-proteomic study on the U251 cell line 3 h after X-ray irradiation (6Gy) and on non-irradiated cells. Our study showed a strong modification of proteoform phosphorylation in response to radiation. We found 453 differentially expressed phosphopeptides (DEPs), with 211 being upregulated and 242 being downregulated. A GO enrichment analysis of DEPs showed a strong enrichment of the signaling pathways involved in DNA damage response after irradiation and categorized them into biological processes (BPs), cellular components (CCs) and molecular functions (MFs). Certain accessions such as BRCA1, MDC1, H2AX, MDC1, TP53BP1 were dynamically altered in our fraction and are highly associated with the signaling pathways enriched after radiation.

## 1. Introduction

Glioblastoma (GBM) is the most common adult primary malignant brain tumor. Despite multi-modal therapy currently available, the prognosis and outcomes of patients with this form of brain cancer are poor [1]. Even in patients receiving aggressive treatment, the median survival is 12–15 month [2]. Its incidence ranges from 0.59 to 3.69 per 100,000 peoples worldwide, and it accounts for over 60% of all adult brain tumors [3]. Notwithstanding its rarity, GBM contributes to 2.5% of the total cancer mortality worldwide due to its poor prognosis [4]. One of the most important hallmarks of GBM is tumor heterogeneity. GBM are known for having an extensively heterogenous histopathology.

Its various forms create a heterogenous tumor microenvironment whose molecular diversities contribute to tumor evolution and treatment resistance [5,6,7].

Several clinical strategies have been implemented in recent years, including surgery, radiotherapy and chemotherapy, but the results did not meet expectations, with a recurrence rate estimated at 90%.

One of the main causes of recurrence is due to the growth of highly invasive tumor cells that infiltrate the surrounding tissue and are not eliminated by standard treatments. Radiotherapy is widely used, predominantly in combination with other cancer treatment modalities. Important advancements in radiotherapy technology have been observed in the past decade. These changes include an upgrade to radiotherapy technology from 2-dimensional whole-brain radiotherapy to 3-dimensional conformal radiotherapy, and more recently to intensity-modulated radiation therapy (IMRT) and volumetric arc radiation therapy (VMAT) [8,9]. In radiotherapy treatment, the Stapp’s regimen (dose fraction 1.8–2.0 Gray over 30 days), which consists of radiotherapy and concomitant chemotherapy with temozolomide, remains the therapy of choice for GBM [4,10]. This fractionation over a span of several weeks improves the radiobiological impact on tumors and, at the same time, preserves healthy tissue. X-rays are a type of ionizing radiation (IR) with biological effects on all cellular components, and one of the main mechanisms of action of radiotherapy is DNA damage to exposed cells. The most important DNA damage are single-strand breaks (SSBs), multiple localized lesions (MLLs) and double-strand breaks (DSBs) [11]. It is estimated that 1 Gy of ionizing radiation induces 1000 SSBs, 40 DSBs and 130 MLLs [11,12]. Thus, despite major advances in radiobiological technology, the prognosis for GBM is far from satisfactory, due to the resistance developed by the tumors.

The response to radiotherapy is not uniform in all patients, and the genetic and molecular variability of GBM makes it difficult to predict. Aggressive growth, early and almost inevitable recurrence and a poor prognosis require novel studies on radioresistance to improve the survival rate and quality of life. Understanding the cellular response mechanism for the DNA Damage Response (DDR) is essential. Indeed, cells possess a cascade of signaling pathways that play important roles in the DDR via post-translational modification (PTM), notably the phosphorylation of a series of interacting proteoforms. When an SSB or DSB occurs in a cell, for example, the phosphorylation of the carboxyl–terminal serine residues of thousands of H2AX molecules is observed; this produces phosphorylated H2AX, or γ-H2AX [13].

Phosphoproteomics profiling with quantitative mass-spectrometry is highly useful for understanding the mechanism of radioresistance, as it can uncover pathways and phosphopeptides involved in this phenomenon. This approach allows researchers to elucidate abnormally activated signaling pathways and to discover therapeutic targets in cancer, as well as cellular pathways and processes [14].

In this study, we aimed to understand the early activity of GBM U251 cell line after irradiation by performing quantitative mass spectrometry-based phosphoproteomics analysis at 3 h post treatment. We also wanted to determine the biological processes that were affected after radiation. The ultimate goal of this study is to reveal potential signaling pathways responsible for promoting DNA damage control and repair that may confer more radioresistant properties to GBM cell lines. Our study provides a new and deeper insight into cellular response to IR and the signaling pathways involved in DDR. A quantitative mass spectrometry-based analysis revealed numerous differentially expressed phosphopeptides (DEPs), related to the following phosphor-proteins, γ-H2AX, pBRCA1, pTOP53BP1 and pMDC1, which are known to play important roles in the DDR after X-ray radiation. Enrichment of these DEPS showed several pathways activated in the early stages after radiation.

## 2. Materials and Methods

### 2.1. Cell Culture

In this study, we used human glioblastoma U251 MG, formerly known and distributed as U-373 MG (ECACC 09063001) [15,16]. We obtained the cell line from CLS Cell Lines Service (GmbH, Eppelheim, Germany). This line was established from a GBM (grade IV astrocytoma) in a 75-year-old patient. It is an adherent cancer cell line characterized by a filamentous morphology [17].

Our cells were cultured in Dulbecco’s modified Eagle’s medium (DMEM, Merck, Darmstadt, Germany) supplemented with 10% fetal bovine serum, 1% antibiotics (Penicillin-Streptomycin Solution, Merck, Darmastadt, Germany) and 2 mM of *L*-glutamine (Merck, Darmstadt, Germany) at 37 °C in a humidified incubator with 5% CO_2_. Cells were used and stored according to the French CODECOH authorization N° DC-2021-4783.

### 2.2. Irradiations

After 72 h of cell culture, the cells were subdivided into two groups of triplicates, for a total of six (6) samples. Irradiations were performed on a Faxitron (CellRad, Precision X-Ray, Madison, CT, USA) using a copper filter, with a tube tension of 129 keV and an intensity of 4.7 mA (corresponding to a dose rate of 1 Gy/min). One of the groups was irradiated with X-ray at 6 Gy and cultured for 3 h after irradiation before stopping. The other cohort constituted the control.

### 2.3. Protein Extraction

The U251 cells were lysed on ice with homemade lysis buffer containing 25 mM of Tris Base, 120 mM of NaCl, 10 mM Triton X and 1 mM EDTA, pH 7.6. This lysis buffer was supplemented with 1 mM of PMSF and protease/phosphatase cocktail inhibitors (Merck, Darmstadt, Germany), before manual lysing using a syringe followed by ultrasonication for 30 s. Manual lysis using an insulin syringe (0.33 mm diameter) followed by sonication steps was repeated twice. The lysate was centrifuged at 12,000× *g* for 30 min and the supernatant was collected into new tubes. Protein concentration was determined using BCA Assay kit (Pierce TM BCA Protein Assay Kit, ThermoScientific, Illkirch, France) according to the manufacturer’s instructions. Protein samples (100 µg) from each condition were adjusted to a final volume of 100 µL using solution A (H_2_O, 0.1% Formic acid) and 2.5 µL of sodium bicarbonate buffer.

### 2.4. Trypsin Digestion and Phosphopeptides Purification

Samples were digested with trypsin/Lys-C in Ammonium Bicarbonate 25 mM pH 7.4 overnight at 37 °C and then dried by a vacuum centrifuge. Then, 300 µL of solution A (H_2_O, 0.1% Formic acid) was added to each digest and loaded onto an equilibration desalting spin column (containing 20 mg resin in a 1:1 water/DMSO slurry). Peptides were bound to the hydrophobic resin under acidic conditions and desalted by washing the columns 3 times with solution A through low-speed centrifugation at 3000× *g* for 1 min. A 300 µL solution with solution B (0.1% Formic acid, 50% ACN) was then applied to each column to elute the bound peptides by centrifugation at 3000× *g* for 1 min, which was carried out twice in succession and then the eluates collected in 2 mL Eppendorf tubes. After elution, the liquid contents of each sample tube were evaporated by dryness vacuum centrifugation.

### 2.5. Phosphopeptide Enrichment

A High Select TM TiO_2_ Phosphopeptide Enrichment Kit (ThermoScientific, Illkirch, France) was applied to enrich the phosphorylated peptides. Briefly, each lyophilized sample was resuspended in 150 µL binding/equilibration buffer, loaded onto the equilibrated spin tip and centrifugated at 1000× *g* for 5 min. Samples were reapplied to the spin tip using the microcentrifuge tube for a second centrifugation at 1000× *g* for 5 min. TiO_2_ spin tips were then successively washed with 20 µL binding/equilibration buffer and 20 µL wash buffer followed by centrifugation at 3000× *g* for 2 min in both stages. A third wash was carried out with 20 µL of solution A with centrifugation at 3000× *g* for 2 min. For column elution, spin tips and adaptors were placed in new collection tubes. Then, 50 µL Phosphopeptide Elution Buffer was loaded into each column and centrifuged at 1000× *g* for 5 min with 1 repetition. Eluates were immediately dried in a speed vacuum concentrator to remove Phosphopeptide Elution Buffer. Eluates were then suspended in 10 µL of solution A for LC-MS analysis.

### 2.6. Liquide Chromatography Tandem Mass Spectrometry (LC-MS) Analysis

LC-MS analyses were performed as previously described [18], using a TIMS-TOF mass spectrometer (Bruker Daltonics, Bruker, Billerica, MA, USA). Database searching and protein abundance were studied using the Peaks XPro software (Peaks studio v10.6, Bioinformatics Solutions Inc., Waterloo, Canada) with FDR 5% for phosphopeptides selection. This FDR was selected in order to maximize the possibility of identifying phosphopeptides, but with a non-negligible probability of selecting false-positive peptides (less than 9% difference with a 1% FDR).

### 2.7. Database Search and Statistical Analysis

Quantification of the proteoform abundance level was carried out using the sum area of the top three unique peptides. A 1.2-fold increase in relative abundance and a *p*-value < 0.05 using Student’s *t*-test from PERSEUS (v 1.6.15.0, Max Planck Institute of Biochemistry, Planegg, Germany) were used to determine those enriched proteins. No further normalization was performed using initial protein abundancy in order to limit any calibration factors. Consequently, the comparison of phosphopeptides between samples is related to differences in phosphorylation and in protein abundance. String Database (https://string-db.org/, accessed on 1 February 2024) was used to construct a protein–protein interaction network. Enrichments in molecular process, cellular process and pathways (KEGG) were performed using ShinyGo (http://bioinformatics.sdstate.edu/go/, accessed on 1 February 2024).

Phospho-proteomic data have been deposited in the iProX public repository database, with the code IPX0009898001 (https://www.iprox.cn, access on 7 November 2024).

## 3. Results

### 3.1. Differentially Expressed Phosphopeptides (DEPs) and Descriptive Results of the Phosphoproteomics Analysis

**Figure 1:**
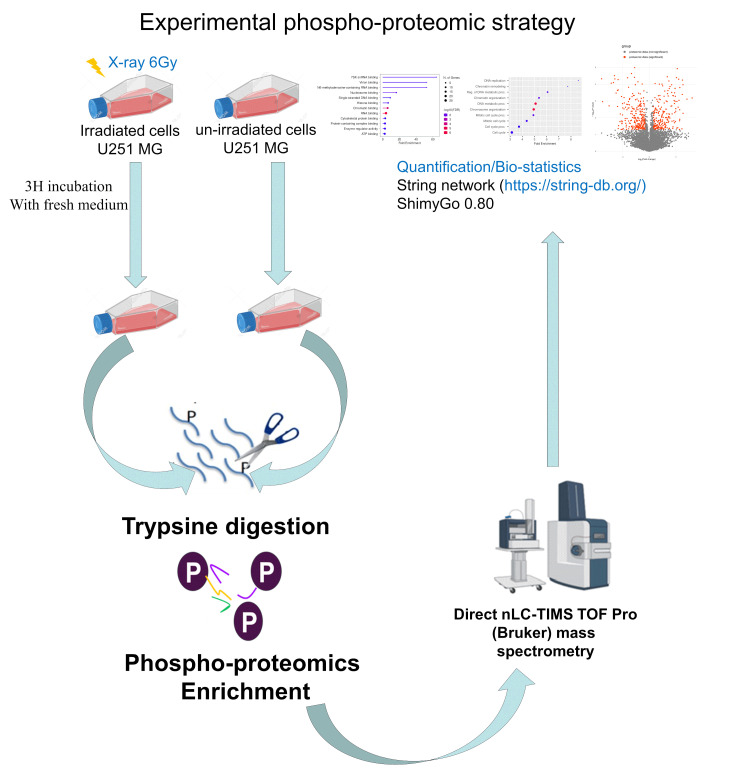
Phosphoproteomics quantitative mass spectrometry-based analysis strategy in GBM U251 cell line after irradiation with X-rays at 6 Gy to discover the early response of the cell line. After irradiation, cells were digested with trypsin for protein extraction and phosphoproteomics enrichment. This step was followed by mass spectrometry and data analysis (https://string-db.org/, accessed on 1 February 2024).

To explore the role of proteoform phosphorylation in the early radiation response, we first established the phosphoproteome landscape using LC-MS/MS quantitative-based phosphoproteomics (Figure 1).

**Figure 2:**
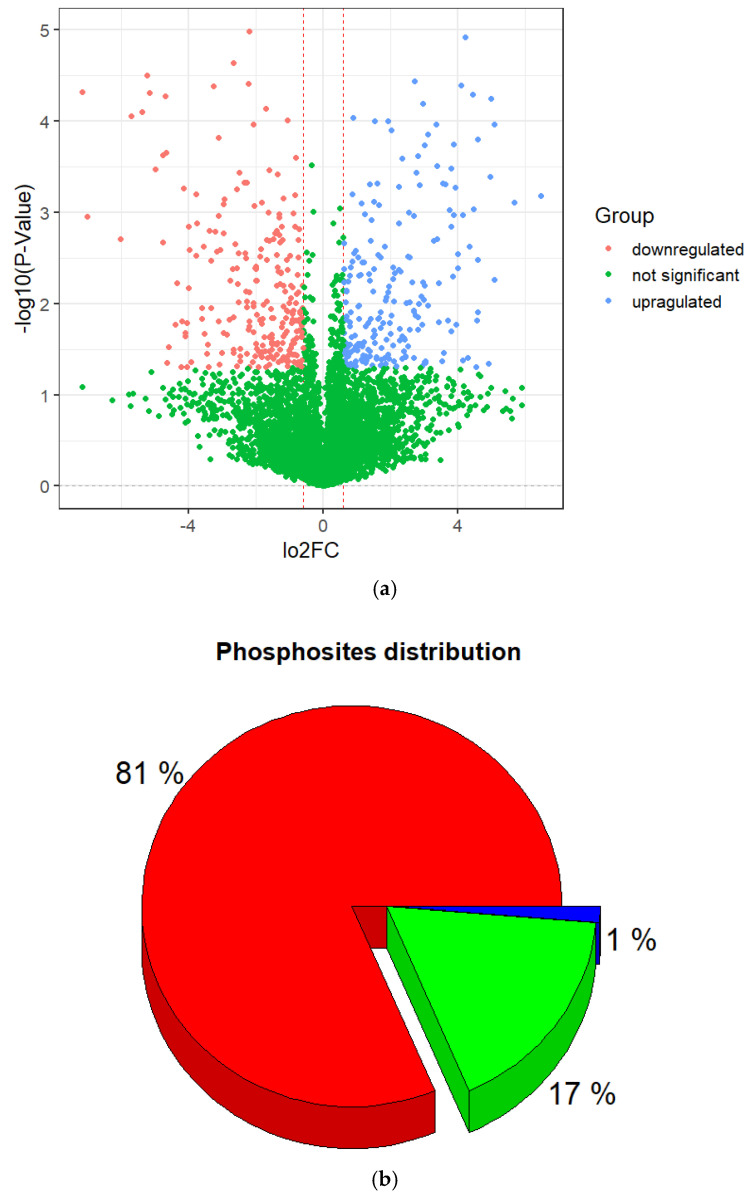
Characterization of identified phosphorylation sites. (**a**) Volcano plot of differentially expressed phosphopeptides. (**b**) Distribution of phosphorylated amino acids in the identified phosphorylation sites.

Normalized log2 transformation ratios were plotted for the phosphopeptides in order to estimate reproducibility and the extent of regulation in different sample groups. The plot below (Figure 2a) indicates major changes in phosphopeptides between irradiated and non-irradiated cells. Here, 5720 phosphorylated peptides were observed at least twice in the three repetitions and identified with a false discovery of less than 1%.

Among them, 453 DEPs were found, including 242 that were downregulated and 211 that were upregulated (Figure 2, Supplementary Table S1) with a *p*-value < 0.05 and log2(FC) ≤ −0.6 and ≥0.6. Also, they were subjected to further descriptive statistical evaluation, which showed that they corresponded to 48311 phosphorylation sites. Among the phosphopeptides, some of them were doubly or triply phosphorylated resulting in a large number of phosphorylation sites. Of the identified phosphorylation sites, the majority occurred at serine (S) residues, accounting for 81% of the sites, followed by threonine (T) 18% and tyrosine (Y) 1% (Figure 2b).

### 3.2. Protein–Protein Interaction (PPI) Network Construction

**Figure 3:**
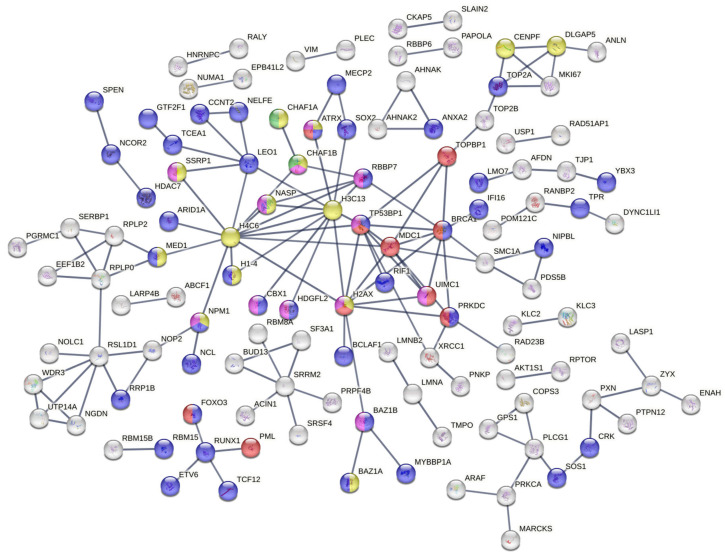
Protein–protein interaction network constructed from differentially expressed proteins. The key hub node of this PPI network shows several clusters of pathways involved in the DDR, which play essential roles in cell survival and radioresistance.

STRING was used to construct a PPI network. PPIs with a confidence higher than 0.9 were selected to ensure the quality of interaction and minimizer false-positive results. The network created from DEPs (Figure 3) showed many clusters related to DNA repair. 

Among them is the signal transduction in response to DNA damage, including TP53BP1 and MDC1 accessions (red color), the regulation of transcription by RNA pol II (Figure 3, blue color) and the CAF-1 complex (green color). Further analysis of the network revealed the representation of a protein–DNA complex (Figure 3, yellow color) biological process and a histone-binding molecular function (Figure 3, purple color). Many other biological process clusters were represented and most of them are related to the response of ionization radiation such as double-strand break repair via nonhomologous end joining (GO:0006303), the positive regulation of cellular component biogenesis (GO:0044089), etc.

### 3.3. Functional Phosphopeptides Analysis

**Figure 4:**
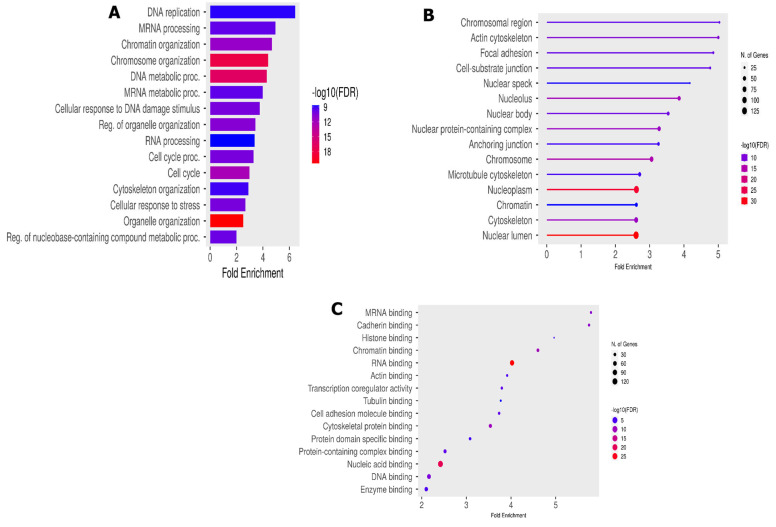
Functional phosphorylation peptides analysis. (**A**) Biological process enrichment; (**B**) cellular component GO enrichment; and (**C**) molecular function GO enrichment analysis.

To understand the biological functions of the 453 modulated phosphopeptides, we assessed the GO enrichment analysis using ShinyGo 0.80. An adjusted *p*-value of <0.05 was set as the cutoff criterion for screening pathway enrichments. The GO enrichment analysis categorized the results into three main categories: biological processes (BPs), cellular components (CCs) and molecular functions (MFs). The top 30 GO enrichment analysis results showed a significant enrichment of GO terms associated with DNA replication (GO:00062260) and mRNA processing (GO:0006281). Alongside this significant enrichment, we notice an important enrichment of DNA repair (GO:0006281) and cell cycle processes (GO:0007049) in the BPs (Figure 4A).

Several accessions were involved in the DNA replication process, such as BRCA1, MCM6, TOPBP1 and TP53BP. These accessions were also shared in other important BP-like cellular responses to DNA damage (GO:0006974) and cellular response to stress (GO:0033554). The enriched cellular components (Figure 4B) mainly include the BRCA1-B complex (GO:0070532), and enriched molecular functions (Figure 4C) include DNA topoisomerase type II (GO:0003918), mediator complex binding (GO0036033) and damage DNA binding (GO:0003684).

## 4. Discussion

Phosphoproteomics analyses offer opportunities to better understand cellular mechanisms in response to X-ray radiation. More than 50% of all cancer patients receive radiotherapy during their course of treatment. However, there is still only limited knowledge about the molecular and cellular effects induced by radiation in GBM. In our study, we compared the acute phosphoproteome response in irradiated and non-irradiated GMB U251 cells line at 3 h after irradiation with 6 Gy of X-rays. The goal was to reveal potential signaling pathways responsible for promoting DNA damage control and repair that may confer radioresistant properties to GBM cancer.

Our study showed a strong modification of proteoform phosphorylation after radiation. The DEPs were closely linked to signaling pathways recognized as playing important roles in genome stabilization and cell survival in response to the effects of ionizing radiation. Accessions such as BRCA1, UIMC1, MDC1 and TP53BP1 were significantly phosphorylated and constituted key hub nodes in the protein–protein network interaction together with H2AX, H3C3 and H4C6 (Figure 3). TP53BP1 is a key regulator in response to IR in other tumor entities such as glioma stem cells [19], lung cancer cells [20] and PDAC cells [21]. Our results thus support other recently published data, which highlighted TP53BP1’s central position in maintaining genome integrity.

MDC1 also plays important roles in the DDR after IR. It has been reported that MDC1 functions as an assembly platform to help localize and maintain signaling and repair factors at and around DSB sites [22]. It is well established that MDC1 mediates the accumulation of many DDR factors in damaged chromatin regions (including the MRN complex, 53BP1, BRCA and ATM [23]). MDC1 is a scaffold protein involved in the early steps of the DDR. It amplifies DNA damage signals by creating a positive feedback loop to concentrate MRN-ATM complexes at the DSB site, which will phosphorylate additional H2AX histone under the form γ-H2AX [24,25]. In this way, MDC1 plays a role as a biomarker for DNA damage, like H2AX, which serves as a reference in radiobiology. In fact, Siddiqui et al. reported in their study that H2AX can also respond to DSBs at a very early stage [26]. When a DNA break occurs, H2AX is phosphorylated at the S139 site, forming γ-H2AX foci after irradiation. In their study, they suggested that this phosphopeptide can be used as a radiosensitizer in cancer treatment. It has been demonstrated that γ-H2AX formation is both a rapid and sensitive response to ionizing radiation. Half-maximal amounts of γ-H2AX are reached by 1 min post-irradiation, and maximal amounts are reached by 10 min [27]. Kue and Yang [28] also suggested that γ-H2AX represents DSBs in a 1:1 ratio and can be used as a biomarker for DNA damage.

Our protein–protein interaction study shows many clusters related to DNA damage repair after ionizing radiation. For instance, we notice signal transduction in response to DNA damage, the regulation of transcription by RNA pol II (blue color) and the CAF-1 complex which is composed of two subunits (CHAF1A and CHAF1B). The CAF1 complex plays an essential role in the replication phases and contributes to genome stability. However, the increased CHAF1B levels are positively correlated with radioresistance by promoting DNA damage repair and cell proliferation. This finding is in line with other studies which show an overexpression of CHA1B in the radioresistance cell line.

The GO enrichment analysis of our DEPs shows a strong enrichment in protein species related to biological process such as DNA replication and mRNA processing, alongside the enrichment of the cell cycle process and the cell cycle itself (Figure 4A). These enriched biological processes are known to be involved in DDR as hallmark of an acute cellular response to ionization radiation [29] and are majority supported by MCM6, POL4 BAZ1A, TP53BP1, BRCA, TOP2B, etc.

In terms of the cellular component category (Figure 4B), we noted a significant enrichment of the BRCA1-B complex, heterochromatin, transcription regulator complex and the molecular functions of DNA topoisomerase type II, mediator complex binding, cytoskeletal protein binding, etc. The formation of BRCA1-B complex by the recruitment of BRIP1 and BACH1 is involved in the DNA damage response in the S phase [30,31]. A defect in the phosphopeptide BCHA1 alters homologous recombination (HR) and leads to a delay in DNA repair [32]. As a result, abnormalities in the BRCA1-B complex could prevent the normal course of the S phase of the cell cycle. The BRCA1-B complex plays a role in DSB repair through HR, although its specific mechanism remains unclear [33] and may be the subject of more advanced studies in the search for new therapeutic targets. It participates as a central component of the macromolecular protein complex and helps to recognize the multiple function of BRCA1 not only in DDR, but also in the transcriptional regulation of genes involved in other cellular process [32,34]. Also, DNA topoisomerase pathways were highly associated with TOP2B and TOP2A. These conserved enzymes are involved in resolving topological problems that arise during a wide range of DNA metabolic processes including DNA replication, cell cycle regulation and cell repair. It was recently demonstrated that TOP2A, particularly its O-GlcNAcylation, promotes malignant breast cancer progression and resistance to Adriamycin (Adm) [35]. TOP2A is highly expressed in many tumor types, such as non-small cell lung cancer, hepatocellular carcinoma, and has proven to be a reliable profile marker, associated with disease progression and poor prognosis [36]. Studies have also revealed that TOP2A is upregulated in medulloblastoma and negatively correlates with the survival time of patients with MB [37,38].

By contrast, several signaling pathways related to cellular stress responses are enriched, such as the cellular response to stress (GO:0033554) and the positive regulation of cellular metabolic process (GO:0031325), as well as biological processes and mitochondrial transcription factor activity (GO:0034246) molecular functions. These pathways are highly associated with the upregulation of FOXO3 (FOXO3a), one of the FOXO transcription factors activated in response to cellular oxidative stress. FOXO3a is a crucial effector of IR-induced apoptosis in response to genotoxic stress [39], caused by high levels of intracellular ROS (reactive oxygen species), e.g. O_2_−, H_2_O_2_ or DNA-damaging hydroxyl ions [40]. One role of FOXO in the stress response is the upregulation of antioxidant phospho-proteoform that mediates the detoxification of ROS and stress resistance that is tightly linked to an increased lifespan [41]. Furthermore, FOXO3a promotes the cell survival pathway and may effectively increase the cellular antioxidant capacity by enhancing the levels of CAT and Prx3 to protect against oxidative stress. In particular, FOXO3a orchestrates differential nuclear/mitochondrial expression programs regulating a variety of cellular metabolism, and ROS scavenging, in response to several stressors. However, our understanding of the FOXO3a in the regulation of ROS is complex, far from being sufficient and requires more investigation. We also identified a major component of the COP9 signalosome (Supplementary Table S1) that consistently shows an increase in phosphorylation and pathway enrichment following radiation treatment in GBM cell lines. To our knowledge, this is the first time that the COP9 signalosome pathway has been linked to the radiation response in GBM. Further investigations are needed to determine the effect of phosphorylation on COP9 signalosome and radiation resistance. The COP9 signalosome is known to regulate numerous cellular and biological process such as the cell cycle, signal transduction and check point repair control [42]. Investigating the role of COP9 and FOXO in the response to irradiation could constitute a new therapeutic target for GBM radioresistance.

Compared to a similar phosphoproteomics study previously carried out on other cell types, we observed an acceptable number of phosphorylation proteoforms. Most of them were found to be enriched at sites in the DDR, in line with current knowledge that the DDR is highly important after ionizing radiation [29]. Furthermore, the observations that multiple signaling pathways are modulated by RIF1 phosphopeptide (Supplementary Table S1) and are related to the DDR in our study are in line with a phosphoproteomics analysis after low- and high-dose exposure in mouse embryonic stem cells [43]. RIF1, a key regulator of TP53BP1, plays a role in the repair of DSBs in response to DNA damage by promoting the NHEJ-mediated repair of DSBs. RIF1 was reported to interact with ATM-phosphorylation TP53BP1 in response to DNA damage [44]. We also identified a deregulated phosphorylation of the well-known GBM driver of the EGFR signaling pathways (GO:0007173), actin cytoskeleton organization and small GTPase-mediated signal transduction (GO:0007264) upon X-ray irradiation. These results are in line with studies demonstrating that these signaling pathways are interconnected by a complex crosstalk mediated by EGFR [45] and a physical interaction of the cytoskeleton regulator Lamellipodin (Lpd) with EGFR [46]. Recently, Moritz et al. showed that the EGFR signaling axis is commonly hyperactive in GBM, depending on Lpd which is also shown to mediate invasiveness, proliferation and radiosensitivity in GBM cells [47]. These findings together emphasize that radiation-induced acute signaling pathways are dominated by DNA repair functions, cell cycle regulation and cell survival/death signals as previously reported [48].

Our data enable the assignment of phosphorylation sites to distinct cellular functions and facilitate further investigations of exact phosphosite functionalities and new targeted pathways in cancer therapeutics.

## 5. Conclusions

According to the results of our study, we have shown early important cellular activity after radiation. An important dynamic and changing pattern of phosphorylation proteomforms was observed. Several phosphopeptides were differentially expressed and a GO enrichment analysis showed that most of them were involved in the cell signaling pathway for DNA damage repair and cell survival with a dynamic proteome complexity. The pathways activated in response to irradiation involved large protein network clusters, and the most important of them contained at least 10 interacting proteins. These protein–protein interactions showed that BRCA and MDC1 play important roles in the upregulation of certain pathways linked to the DNA damage response, including DNA replication, signal transduction to the DNA damage response and the regulation of DNA repair pathways. Other mechanisms involved in the cellular stress response were also enriched. These pathways open up new possibilities for combating oxidative radioresistance and reducing ROS-scavenging proteins during radiotherapy treatment for GBM.

## Data Availability

Raw data are available in the iProX public repository database https://www.iprox.cn (access on 7 November 2024)-IPX0009898001.

## Supplementary Materials

The following supporting information can be downloaded at: https://www.mdpi.com/article/10.3390/proteomes13010001/s1, Table S1: List of protein accessions with differential phosphorylation, including peptide sequences and *p* values.

## Abbreviations

ATM: Ataxia telangiectasia mutated; BACH1: BTB Domain and CNC Homolog 1; BAZIA: Bromodomain Adjacent to Zinc Finger Domain 1A; BP: Biological process; BRCA1: Breast cancer gene 1; CAF: Chromatin assembly complex; CAT: Catalase; CC: Cellular component; CHAF1A: Chromatin assembly factor 1 subunit a; CHAF1B: Chromatin assembly factor 1 subunit b; DDR: DNA damage repair; DEP: Differentially expressed phosphoproteins; DSB: Double strand break; EGFR: Epithelial growth factor receptor; FC: Fold change; FOXO: Forkhead box O; FOXO3: Forkhead box O3; GBM: Glioblastoma; GO: Gene ontology; H2AX: H2A. X Variant Histone; H_2_O_2_: Hydrogen peroxide; H3C3: H3 clustered histone 3; H4C6: H4 clustered histone 6; HR: Homologous recombination; IMRT: Intensity-modulated radiation therapy; IR: Ionization radiation; KEGG: Kyoto encyclopedia of genes and genomes; LC-MS: Liquide chromatography mass spectrometry; MCM6: Minichromosome maintenance complex component 6; MDC1: Mediator of dna damage checkpoint 1; MF: Molecular function; MLL: Multiple localized lesions; mRNA: Messenger ribonucleic acid; NHEJ: Non-homologous end joining; PDAC: Pancreatic ductal adenocarcinoma; PMSF: Phenylmethylsulfonyl fluoride; POL4: DNA-directed DNA polymerase iv; PPI: Protein–protein interaction; PRX3: peroxiredoxin 3; PTM: Post-translational modification; RIF1: Replication timing regulatory factor 1; RNA: Ribonucleic acid; ROS: Reactive oxygen species; SSB: Single strand breaks; TFA: Trifluoroacetic acid; TiO_2_: Titan Dioxyde; TOF: Times of flight; TOP2A: DNA topoisomerase ii alpha; TOP2B: DNA topoisomerase ii beta; TOPBP1: Topoisomerase II binding protein 1; TP53BP1: Tumor protein p53 binding protein 1; UIMC1: Ubiquitin interaction motif containing 1; VMAT: Volumetric arc radiation therapy.
